# Barriers and Facilitators to Implementing Cognitive Stimulation and Reminiscence Therapy for Dementia in Care Homes: Systematic Review

**DOI:** 10.1002/gps.70124

**Published:** 2025-07-12

**Authors:** Emily Fisher, Isobel Chick, Jane Fossey, Aimee Spector

**Affiliations:** ^1^ Research Department of Clinical Educational and Health Psychology University College London London UK; ^2^ Sheffield Institute for Translational Neuroscience University of Sheffield Sheffield UK; ^3^ Department of Psychology University College London London UK; ^4^ Faculty of Health and Life Sciences University of Exeter Exeter UK

**Keywords:** care homes, consolidated framework for implementation research, dementia, implementation, long‐term care, nursing homes, psychosocial intervention, systematic review

## Abstract

**Objectives:**

Psychosocial interventions play a vital role in addressing the complex needs of people with dementia in care homes. Cognitive stimulation and reminiscence therapy are recommended by the UK National Institute for Health and Care Excellence to support the cognition, independence, and wellbeing of people with dementia, and crucially, they can be delivered by care home staff or non‐specialist interventionists. This review aims to explore factors that influence the implementation of cognitive stimulation and reminiscence therapy for people with dementia delivered by staff in care homes.

**Methods:**

Ten electronic databases were searched between 2000 and April 2024. Two reviewers systematically appraised the studies for inclusion using pre‐specified criteria and their quality using the Critical Appraisal Skills Programme (CASP) and Mixed Methods Appraisal Tool (MMAT) checklists. Data was analysed thematically using a deductive approach based on the updated Consolidated Framework for Implementation Research (CFIR), and findings were synthesised narratively.

**Results:**

Nine studies were included; three focussed on reminiscence therapy, and six on cognitive stimulation. All interventions were delivered in care homes by care home staff. Many studies were excluded because a research team member delivered the intervention. Overall, the quality of the studies was low. Key facilitators to implementation were the availability of standardised manuals or resources, the adaptability of interventions, and staff training and support. Barriers included a lack of staff time and availability and a lack of perceived support from care home management. Most studies collected quantitative outcomes, and a minority collected qualitative information about implementation experiences and perceptions of the intervention. No studies collected qualitative data from people with dementia or their carers.

**Conclusions:**

The review highlights the field's reliance on research staff to deliver interventions rather than training and involving care home staff in evaluating interventions. Additionally, there is a lack of qualitative data from people with dementia and their families regarding their views, preferences, and experiences related to participating in psychosocial interventions in care homes. There is a pressing need for high‐quality evidence on the implementation of interventions for dementia, which involves collaboration, consultation and co‐design with those who will deliver the intervention routinely and the people with dementia who will receive the intervention.

**Trial Registration:**

CRD42022313337

## Summary

- Key implementation facilitators were standardised manuals, adaptable interventions, and staff training and support.
- Key barriers included insufficient staff time and perceived support from care home management.
- Many studies depend on research staff to deliver interventions instead of training and involving care home staff.
- There was limited qualitative data from people with dementia and their families on psychosocial interventions in care homes.

## 1 Introduction

Dementia affects over 55 million people worldwide [1] and has a significant negative impact on cognition, independence, and wellbeing [2]. People with dementia have complex care needs, which become greater as the condition progresses, often resulting in the need for a move to a care home or nursing home. [3]. Recent estimates suggest that around 70% of people living in care homes have dementia or severe memory problems [4].

The prevalence of depressive and behavioural symptoms in people with dementia in care homes is high [5, 6], with associated impact on residents' quality of life [7, 8]. Additional concerns relate to the overprescription of antipsychotic medication to treat neuropsychiatric symptoms [9, 10], and there are ongoing efforts to address unmet needs through non‐pharmacological interventions [11, 12].

Non‐pharmacological or psychosocial interventions can play a pivotal role in addressing the complex needs of people with dementia in care home settings. These interventions use a wide range of approaches to maximise cognitive functioning, promote independence in day‐to‐day activities, and improve the quality of life for people with dementia, and can include cognitive stimulation and reminiscence therapy [11, 12, 13].

### 1.1 Cognitive Stimulation

Cognitive stimulation is a psychosocial intervention comprised of activities which aim to stimulate cognition and memory through tasks, including group discussions, puzzles, music and creative arts [14]. A systematic review of cognitive stimulation programmes reported benefits to cognition, self‐reported quality of life, communication and social interaction [14].

Many cognitive stimulation programmes have been developed. The first was group Cognitive Stimulation Therapy (CST), which is comprised of 14 twice‐weekly, themed sessions which are underpinned by 18 key principles, including encouraging new ideas and associations, consistency between sessions, and focussing on opinions rather than facts [15]. CST is cost‐effective in the UK [16] and is widely implemented as a post‐diagnostic intervention in NHS memory clinics [17, 18]. It was initially tested and developed in care homes [15], but the extent of its implementation in this setting is unclear.

Other structured cognitive stimulation programmes have been developed and evaluated, including 'MAKS', which consists of motor stimulation, activities of daily living and cognition and social communication [19]. However, to date, they have been less widely adopted [14].

### 1.2 Reminiscence Therapy

Reminiscence therapy involves discussing past events and experiences to evoke memories, stimulate mental activity and improve wellbeing. It can occur in a structured group setting or individually and often requires props and prompts, including images, videos and objects [20]. A recent review suggests that group reminiscence therapy is associated with improved communication, and the impact on quality of life appears most promising in care home settings [20].

### 1.3 Implementation Gap

Despite the evidence base for these psychosocial interventions and recommendations for their use, successful implementation in care homes remains a challenge [11]. Understanding the barriers and facilitators to implementation is crucial for delivering psychosocial interventions and ultimately improving outcomes for people with dementia in care homes.

Cognitive stimulation and reminiscence therapy can be delivered by non‐specialists, as opposed to interventions such as cognitive rehabilitation, music therapy, and occupational therapy, which need Health and Care Professions Council‐registered therapists [21, 22, 23].This provides an opportunity for care home staff to deliver such interventions.

A 2013 UK government review presented the need for improved training and support of healthcare and care assistants in care homes [24]. The same report highlighted that many care home staff value the relational aspects of working with care home residents [24]. More than 10 years on, a report by Alzheimer's Society highlighted that only 29% of care staff have had any basic dementia training [25, 26], despite its associated impact on residents' quality of life, increased staff job satisfaction and savings across the health and care system [26].

### 1.4 Objectives

The main objective of the review is to examine factors influencing the implementation of cognitive stimulation and reminiscence therapy for people with dementia, specifically when these therapies are provided by staff in care homes.

Previous reviews have examined factors influencing the implementation of a broader range of psychosocial interventions in care homes [11, 27, 28], with dates of inclusion until 2011, 2016 and 2018 respectively. This review specifically focuses on cognitive stimulation approaches (not limited to CST) and reminiscence therapy.

Both cognitive stimulation and reminiscence therapy are recommended by the National Institute for Health and Care Excellence (NICE) to promote cognition, independence, and wellbeing for individuals with dementia [17]. These therapies can be delivered by care home staff in a group setting. Their inclusion in NICE guidelines demonstrates their efficacy and cost‐effectiveness, and as a result we did not collect or report data on these aspects.

We believe that staff training is a crucial element for the successful and long‐term implementation of these therapies, so we focus on studies where the interventions were delivered by care home staff.

## 2 Methods

### 2.1 Design

This is a systematic review using thematic analysis and narrative synthesis. The review is reported following PRISMA guidelines [29] (see Appendix 1 for the PRISMA checklist). We registered the review at PROSPERO (registration number CRD42022313337) and searched PROSPERO before registration to ensure no similar reviews were in progress.

### 2.2 Eligibility Criteria

The eligibility criteria were developed using the population, intervention, control, outcomes, and study design) (PICOS) framework (see Table 1). Studies that included multiple populations (e.g., people living in care homes and community settings) were only included if the data were reported separately.

**TABLE 1:** Eligibility criteria.

*Inclusion criteria* **Population:** People with dementia living in care homes **Intervention**: Structured psychosocial interventions delivered directly to people with dementia by staff in care homes (group cognitive stimulation and reminiscence therapy) **Outcome:** Data relating to factors that influence or inhibit the implementation of psychosocial interventions in care homes **Comparator:** No restrictions **Study design:** Qualitative studies, process evaluations, quantitative with a control group (pre‐post studies/randomised controlled trials)
*Exclusion criteria* **Population**: People with dementia in the community, family caregivers of people with dementia in care homes **Intervention**: Staff training and care practice interventions (such as person‐centred care or training to manage behavioural symptoms of dementia) **Outcome**: Not applicable **Comparator**: Not applicable **Study design**: Single case studies, conference abstracts, study protocols, systematic reviews.

### 2.3 Search Strategy

We used keywords to develop a search strategy for the following concepts: dementia, psychosocial intervention, and care homes. We populated search strings using keywords from previous systematic reviews [27, 30] and through consultation with a subject expert librarian. The full electronic search strategy for MEDLINE is available in Appendix 2.

We searched nine databases in April 2022: Applied Social Sciences Index and Abstracts (ASSIA), British Nursing Index (BNI), Cumulative Index to Nursing and Allied Health Literature (CINAHL), Cochrane Central Register of Controlled Trials (CENTRAL), Embase, Healthcare Management and Information Consortium (HMIC), MEDLINE, PsycINFO and Social Practice and Policy (SPP). We re‐ran the searches in April 2024 for all but BNI, which was no longer available due to a cyber‐attack on the database. We hand‐searched the references of 11 systematic reviews [31, 32, 33, 34, 35, 36, 37, 38, 39, 40], which were identified through the above search and additional searching of the Cochrane Database of Systematic Reviews (CDSR).

The search did not restrict language or date. However, papers before 2000 and those not in English were excluded upon screening. The year 2000 was chosen as a cut‐off to exclude work that may be outdated in the context of psychosocial interventions.

### 2.4 Study Selection

We imported the search results into EndNote and deduplicated the records before importing them into Rayyan for screening. Two researchers (EF and IC) screened the title and abstract against inclusion criteria. Both screened a random sample of 30 to ensure they were similarly approaching the task and to test out applying the eligibility criteria. EF continued to screen all titles and abstracts, and IC screened a random sample of 46%. Any discrepancies were resolved through discussion and consultation with a third researcher (AS).

The full papers identified in the initial screening were screened and excluded according to the exclusion criteria. EF screened all eligible full texts, and IC independently screened 41%. Where information about eligibility criteria was missing from the paper, we contacted the corresponding author for this information.

### 2.5 Data Extraction

A data extraction tool was developed in Microsoft Excel. Data extracted included study design and aim, intervention, setting, interventionist, frequency of intervention, number of care homes/nursing homes, and country. The included papers were imported into NVivo 14 to extract data related to implementation. One reviewer undertook the data extraction, with a second reviewer checking a proportion and resolving discrepancies through discussion.

### 2.6 Data Analysis and Synthesis

We analysed data thematically, using a deductive approach based on the Updated Consolidated Framework for Implementation Research (CFIR) [41]. This is a determinant framework which incorporates 48 constructs across five domains related to implementation.

1. intervention characteristics (e.g. the intervention's core and adaptable components)
2. outer setting (e.g. external partnerships and financing)
3. inner setting (e.g. available organisational resources and staffing)
4. roles and characteristics of individuals involved in implementation (including their need for the intervention, capability, availability, and motivation to be involved based on the COM‐B model) [42].
5. implementation processes (e.g. planning and tailoring strategies).

We considered that some data may not fit within the CFIR, so we coded these inductively. We carried out additional extraction and narrative synthesis of implementation outcomes based on Proctor and colleagues' 2011 taxonomy, comprised of acceptability, adoption, appropriateness, feasibility, fidelity, implementation cost, penetration, and sustainability [43].

We coded text in NVivo to the relevant CFIR construct or implementation outcome. Data from the methods, results, and discussion sections were coded to capture broader reflections on intervention implementation. We narratively synthesised common themes across studies to highlight barriers and facilitators to implementation. This was aligned with the guidance provided by Popay et al. (2006), which provides specific guidelines for reviews focussed on implementing interventions [44].

### 2.7 Quality Appraisal

All papers were appraised independently by two researchers (EF and IC). We used the relevant Critical Appraisal Skills Programme (CASP) checklists for randomised controlled trials (RCTs) [45] and qualitative studies [46]. We did not use the final section of the RCT checklist for the practical application of the findings. We used the Mixed Methods Appraisal Tool (MMAT) for mixed methods studies [47]. We assessed characteristics, including the appropriateness of methodology and rigour of data analysis and reporting. Discrepancies between the two researchers were discussed, and a consensus was reached with input from a third researcher. No studies were excluded based on their appraised quality, but quality was considered when interpreting the results.

## 3 Results

### 3.1 Study Selection

**FIGURE 1:**
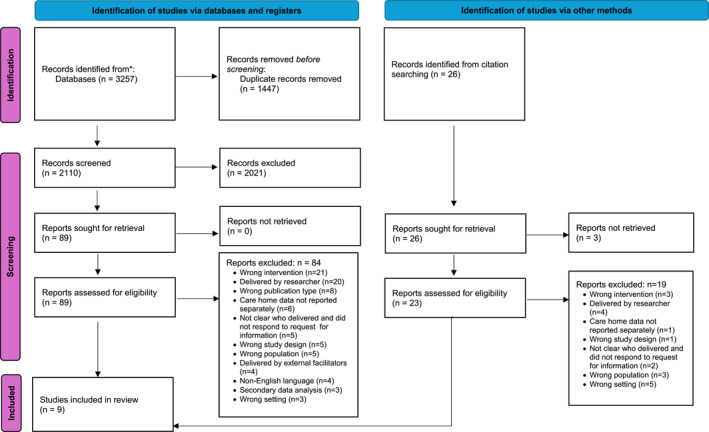
Diagram of inclusion.

A total of 2136 papers were screened by title and abstract, of which 112 full‐text articles were assessed. Nine articles were included in the review and narrative synthesis. See Figure 1 for a summary of inclusion and exclusion [29].

### 3.2 Study Characteristics

All interventions were delivered in care homes by care home staff. Three studies explored the use of reminiscence therapy [48, 49, 50]. Six studies explored the use of cognitive stimulation [19, 51, 52, 53, 54, 55]. Specifically, cognitive stimulation interventions included MAKS [19, 53, 54], CST [52, 55] and a multi‐modal intervention combining reality orientation, reminiscence therapy and daily activities following brain‐activating rehabilitation (BAR) principles ‐ pleasant atmosphere, communication, praising, social role, and supportive care [51]. Seven studies were RCTs [19, 48, 49, 51, 53, 54, 55], one was a service evaluation [52] and one was a formative evaluation [50]. Tables 2 and 3 gives a summary of the included studies.

**TABLE 2:** Study characteristics.

Reference	Intervention	Study design	Aim	Setting (*n*)	Population (*n*)	Interventionist (*n*)	Duration/frequency	Country
Clark, 2017 [50]	Sports‐based reminiscence therapy	Formative evaluation	Describe findings from pilot and follow‐on study where care assistants were trained to use sporting memories	Care homes (15)	Residents with dementia (not specified)	Care assistants (not specified)	Not specified	UK
Coen, 2011 [55]	CST	Randomised controlled trial	Investigate effectiveness of CST on people with dementia	Long‐term care and nursing home (3)	Residents with dementia (27)	Occupational therapists and activity coordinators (not specified)	Seven twice‐weekly sessions	Ireland
Graessel, 2011^a^ [19]	MAKS	Randomised controlled trial	Investigate effectiveness of MAKS on people with dementia	Nursing homes (5)	Residents with dementia (98)	Care home staff (not specified)	2‐h sessions, 6 days a week for 12 months	Germany
Gudex, 2010 [48]	Reminiscence therapy	Randomised matched intervention study	Investigate the effects of integrating reminiscence into daily care for residents and staff	Nursing homes (10)	Residents with and without dementia (348)	Nursing home staff (353)	12‐month duration. Frequency not specified	Denmark
Kratzer, 2022 [54]	MAKS	Cluster randomised controlled trial	Investigate effectiveness of MAKS on people with severe dementia	Nursing homes (26)	Residents with severe dementia (152)	Care home staff (not specified)	3 times weekly over 6‐month	Germany
Lök, 2019 [49]	Reminiscence therapy	Randomised controlled trial	Investigate effectiveness of reminiscence therapy on people with dementia	Care home (1)	Residents with Alzheimer's disease (60)	Nurse from care home (1)	Eight weekly sessions	Turkey
Luttenberger, 2012^a^ [53]	MAKS	Randomised controlled trial	Investigate effectiveness of MAKS on people with dementia	Nursing homes (5)	Residents with dementia (139)	Care home staff (not specified)	6 months, 2 h per day, 6 days a week	Germany
Streater, 2016 [52]	CST	Service evaluation	Train and offer outreach support to care home staff to implement CST	Care homes (12)	Residents with dementia (not specified)	Care home staff (46)	Seven twice‐weekly sessions	UK
Yamagami, 2012 [51]	Brain‐activating rehabilitation (BAR)	Randomised controlled trial	Investigate effectiveness of BAR on people with dementia	Care home (1)	Residents with dementia (54)	Care home staff (41)	12 twice weekly sessions	Japan

^a^ Articles from the same study.

**TABLE 3:** Summary of qualitative and quantitative data collected.

Reference	Qualitative data		Quantitative outcomes	
	Staff	People with dementia	Staff	People with dementia
Clark, 2017 [50]	Surveys of staff after training and at project end, and interviews with a lead person from the care home exploring attitudes to and experiences of implementation	—	—	—
Coen, 2011 [55]	—	—	—	Measures of cognition (MMSE, ADAS‐Cog), quality of life (QOL‐AD), behaviour (CAPE‐BRS), dementia severity (CDR) and anxiety (RAID). Ratings by group leader on participants' interest, enjoyment of communication, and mood in each session.
Graessel, 2011^a^ [19]	—	—	—	Measures of cognition (ADAS‐Cog), activities of daily living (EADL and depression (NOSGER)
Gudex, 2010 [48]	Questionnaire and interviews with staff members in the intervention group to explore attitudes to and experiences of implementation	—	Burnout (MBI‐HSS), work satisfaction (SNCW), self‐rated health (SF12‐v2)	Agitation (CMAI), quality of life (ADRQL), general functioning and activities of daily living (GBS), and cognition (MMSE, SIB‐S)
Kratzer, 2022 [54]	—	—	—	Neuropsychiatric symptoms (NPI‐NH), quality of life (QUALIDEM), cognition (ADCS‐ADL‐sev)
Lök, 2019 [49]	—	—	—	Cognition (MMSE), depression (Cornell), quality of life (QoL‐AD)
Luttenberger, 2012^a^ [53]	—	—	—	Measures of cognition (ADAS‐Cog), activities of daily living (E‐ADL and depression (NOSGER)
Streater, 2016 [52]	—	—	Perceptions about people with dementia (ADQ), dementia knowledge (DKAS), Competence (SCIDS), learning transfer (LTSI)	—
Yamagami, 2012 [51]	Two questions exploring perceived changes in people with dementia and changes to daily care provided	—	—	Dementia severity (MOSES, CDR), cognition (HDS‐R, TMT‐A)

Abbreviations: ADAS‐Cog, Alzheimer's Disease Assessment Scale—Cognitive subscale; ADCS‐ADL‐sev, Alzheimer's Disease Cooperative Study Activities of Daily Living Scale—severe dementia; ADRQL, Alzheimer Disease Related Quality of Life; ADQ, Approaches to Dementia Questionnaire; CAPE‐BRS, Clifton Assessment Procedures for the Elderly‐Behaviour Rating Scale; CDR, Clinical Dementia Rating; CMAI, Cohen‐Mansfield Agitation Inventory; DKAS, Dementia Knowledge Assessment scale; E‐ADL, Erlangen Test for Activities of Daily Living; GBS, Gottfries‐Bråne‐Steen scale; HDS‐R Hierarchic Dementia Scale‐Revised; LTSI, Learning Transfer System Inventory; MBI‐HSS, Maslach Burnout Inventory ‐ Human Services Survey; MMSE, Mini‐Mental State Examination; MOSES, Multidimensional Observation Scale for Elderly Subjects; NOSGER, Nurses' Observation Scale for Geriatric Patients; NPI, Neuropsychiatric Inventory—Nursing Home Version; QOL‐AD, Quality of Life in Alzheimer's Disease; QUALIDEM, Quality of Life for People with Dementia; RAID, Rating Anxiety In Dementia; SCIDS, Sense of Competence in Dementia Care Staff; SF12‐v2, Short Form Health Survey version 2; SIB‐S, Severe Impairment Battery ‐ Short Form; SNCW, Satisfaction with Nursing Care and Work Assessment; TMT‐A, Trail Making Test Part A.

^a^ Articles from the same study.

### 3.3 Study Quality

The included studies varied in quality but tended towards lower quality. Appendix 3 provides a detailed overview of study quality. Seven studies were assessed according to the CASP RCT checklist [19, 48, 49, 51, 53, 54, 55]. On average, 49% of the criteria were met. We identified straightforward research questions and study protocols, but highlighted issues with randomisation, nonblinding, and small sample sizes. The review did not focus on evaluating the efficacy of the interventions. Therefore, the low quality of these RCTs does not directly affect our findings related to implementation. However, it does indicate a potential trend in the field towards lower‐quality research.

More relevant to our focus on exploring implementation issues are qualitative and mixed‐methods studies. Three studies were appraised according to the CASP qualitative checklist. One was a qualitative paper [50], and two were RCTs with qualitative components [48, 51]. On average, 41% of the criteria were met. There were clear statements of aims and findings, but issues with the rigour and reporting of data analysis, failure to consider the relationship between the researcher and participants, and failure to address the risk of bias from sampling. We used the MMAT for one study to assess the quality of a study with quantitative, non‐randomised, and descriptive elements [52], where 30% of the criteria were met. Issues related to not accounting for confounders in quantitative analyses, a lack of sample representativeness and a high risk of nonresponse bias.

### 3.4 Intervention Deliverer

In all nine studies, the intervention was delivered by care home staff (including care assistants, occupational therapists, activity coordinators, and nurses).

Many studies did not explicitly report who delivered the intervention. I contacted the authors of 15 full texts that met the inclusion criteria but did not specify who delivered the intervention. Seven did not respond and were excluded. Eight responded; researchers delivered the intervention in five studies, which were subsequently excluded, and care home staff delivered the intervention in three studies, which were included. A large proportion of full‐text articles (22%; *n* = 24) met all the inclusion criteria apart from the intervention deliverer belonging to the research team. Eight of these studies investigated cognitive stimulation [56, 57, 58, 59, 60, 61, 62, 63], 14 reminiscence therapy [64, 65, 66, 67, 68, 69, 70, 71, 72, 73, 74, 75, 76, 77], and two explored both [78, 79].

### 3.5 CFIR Constructs Associated With Implementation

Table 4 shows the spread of constructs across the studies. The most widely reported constructs related to the design of the intervention and materials, partnerships and connections between the care home and research teams, intervention resource availability and access to training.

**TABLE 4:** CFIR constructs across individual studies.

CFIR domains and constructs	Clark, 2017 [50]	Coen, 2011 [55]	Graessel, 2011 [19]	Gudex, 2010 [48]	Kratzer, 2022 [54]	Lök, 2019 [49]	Luttenberger, 2012 [53]	Streater, 2016 [52]	Yamagami, 2012 [51]	Number of studies per construct
I. INNOVATION										
A. Innovation source										
B. Innovation evidence‐base										
C. Innovation relative advantage										
D. Innovation adaptability	X			X					X	**3**
E. Innovation trialability	X									**1**
F. Innovation complexity										
G. Innovation design	X	X	X	X	X	X	X	X	X	**9**
H. Innovation cost		X	X							**2**
II. OUTER SETTING										
A. Critical incidents					X					**1**
B. Local attitudes										
C. Local conditions										
D. Partnerships and connections	X		X	X	X		X	X	X	**7**
E. Policies and laws		X	X							**2**
F. Financing	X		X	X	X		X	X		**6**
G. External pressure										
1. Societal pressure										
2. Market pressure										
3. Performance‐measurement ‐pressure										
III. INNER SETTING										
A. Structural characteristics										
1. Physical infrastructure										
2. IT infrastructure	X									**1**
3. Work infrastructure	X			X	X					**3**
B. Relational connections	X									**1**
C. Communications										
D. Culture										
1. Human equality‐centeredness										
2. Recipient‐centeredness	X			X						**2**
3. Deliverer‐centeredness										
4. Learning‐centeredness										
E. Tension for change										
F. Compatibility	X	X	X				X			**4**
G. Relative priority	X			X						**2**
H. Incentive systems										
I. Mission alignment										
J. Available resources										
1. Funding				X						**1**
2. Space										
3. Materials and equipment	X		X	X	X	X	X	X	X	**8**
K. Access to knowledge and information	X		X	X	X		X	X	X	**7**
IV. INDIVIDUALS										
A. High‐level leaders				X						**1**
B. Mid‐level leaders										
C. Opinion leaders										
D. Implementation facilitators								X		**1**
E. Implementation leads				X	X					**2**
F. Implementation team members										
G. Other implementation support	X			X						**2**
H. Innovation deliverers	X			X		X			X	**4**
I. Innovation recipients A. Need B. Capability C. Opportunity D. Motivation	X X X	X X	X X	X X X X	X	X		X	X X X	**4** **4** **4** **5**
V. IMPLEMENTATION PROCESS										
A. Teaming										
B. Assessing needs										
1. Innovation deliverers										
2. Innovation recipients			X	X					X	**3**
C. Assessing context										
D. Planning										
E. Tailoring strategies										
F. Engaging										
1. Innovation deliverers										
2. Innovation recipients										
G. Doing	X									**1**
H. Reflecting and evaluating										
1. Implementation	X	X	X	X	X		X	X	X	**8**
2. Innovation	X	X	X	X	X	X	X	X	X	**9**
I. Adapting	X									**1**
**Number of constructs per study**	**20**	**7**	**12**	**19**	**10**	**5**	**7**	**8**	**11**	

*Note:* The count of constructs is based on those labelled alphabetically. Sub‐constructs labelled numerically are aggregated in their parent construct.

The number of CFIR constructs identified within each paper ranged from 5 to 20, with a median of 10. The total number of constructs identified in the review was 30 (out of a total of 48).

#### 3.5.1 Innovation

This domain relates to features of cognitive stimulation and reminiscence therapy that may affect the success of implementation in care homes.

##### 3.5.1.1 Design

All studies reported using manuals or standardised materials to support intervention delivery and implementation. Studies of MAKS [19, 53, 54] and CST [52, 55] reported the use of a standardised manual, whilst two studies of reminiscence therapy reported the use of standardised resources, including reminiscence boxes and printed and digital publications distributed across multiple care homes [48]. The study of BAR reported the use of standardised guidelines, which the intervention deliverers studied and followed [51].

##### 3.5.1.2 Adaptability

Whilst standardised materials were highlighted as necessary, three of the studies reported the importance of the adaptability of the intervention [48, 50, 51]. The formative evaluation of sports‐based reminiscence therapy revealed facilitators appreciated the intervention's flexibility regarding group size and session length. Additionally, whilst the programme is centred around sports, it does still facilitate the sharing of non‐sporting memories, which attracts individuals who may not have a strong interest in sports [50]. Two studies of reminiscence therapy highlighted the importance of adapting contents to the sessions according to participants' life histories [48, 51]. In a multi‐site trial of reminiscence therapy, the authors highlighted that the opportunity to adapt and tailor the intervention to individual nursing homes was missed, as delivery needed to be consistent across the trial sites [48].

##### 3.5.1.3 Cost

Two studies specifically mentioned the cost of the intervention [19, 55]. One study of MAKS reports the costs of therapy per day as primary data [19], whilst authors of a study of CST reflect that its proven cost‐effectiveness is an enabler of implementation [55].

##### 3.5.1.4 Outer Setting

The outer setting refers to factors external to the care home that affect intervention implementation, such as those related to the care system, external partners, or the local community.

##### 3.5.1.5 Critical Incidents

One study experienced disruption in implementing the intervention in the care home due to unexpected events. One study of MAKS occurred during the COVID‐19 pandemic, severely hindering the intervention's delivery [54].

##### 3.5.1.6 Partnerships and Connections

Seven studies mentioned specific links between the care home and a research team, through training or outreach support [19, 48, 50, 51, 52, 53, 54], but by definition of their involvement in the research studies, all care homes were partnered with a research team.

In the study of sports‐based reminiscence therapy, participating care homes joined a network of other homes, which gave teams access to training, learning set meetings and an online knowledge exchange forum [50].

In three studies of MAKS, CST and reminiscence therapy, all participating care homes had support and formalised follow‐up visits from a research team member [19, 48, 52].

##### 3.5.1.7 Financing

Six studies report funding from external bodies [19, 48, 50, 52, 53, 54]. These were generally grants for the research project, but the grant for sports‐based reminiscence was from Skills for Care, which had a more practical focus on implementing and developing a network of care homes [50].

##### 3.5.1.8 Policies and Laws

CST is recommended in NICE guidelines, and the authors of one study referred to this as an enabler of wider implementation [55]. One study of MAKS advocated for the use of non‐drug treatments and highlighted opportunities for delivery within supplementary care services, which are a standard of the German health system [19].

##### 3.5.1.9 Critical Incidents

One study experienced disruption in implementing the intervention in the care home due to unexpected events. One study of MAKS occurred during the COVID‐19 pandemic, severely hindering the intervention's delivery [54].

#### 3.5.2 Inner Setting

The inner setting describes the setting in which the intervention is implemented. In all nine studies, the inner setting is a care home or nursing home.

##### 3.5.2.1 Available Resources—Materials and Equipment

Eight studies referred to the availability of materials and equipment within the care home. In the three studies of MAKS and one study of CST, all care homes had access to the standardised manual, usually through provided by the research team [19, 52, 53, 54]. No studies reported on the cost of the manual required to deliver the intervention. The study of BAR used old‐fashioned tools and textbooks [52].

The three studies of reminiscence therapy highlighted the resources required to run reminiscence therapy, including photographs, household goods, reminiscence boxes and printed and digital publications [48, 49, 50]. While the materials provided to care homes by one study organiser were considered sufficient for reminiscence work to begin [50], another study reported that the standardised reminiscence boxes needed to be supplemented with more varied materials [48].

##### 3.5.2.2 Available Resources—Funding

In contrast to the majority of studies which received external funding, one study of reminiscence therapy was self‐financed by nursing homes that provided staff coverage whilst the permanent nursing staff attended the training course [48]. The authors reflected that this could have resulted in a self‐selection bias of care homes that could cover these costs.

#### 3.5.3 Access to Knowledge and Information

Seven studies reported that care homes were provided with training for staff to deliver the intervention [19, 48, 50, 51, 52, 53, 54]. The research team delivered these, which ranged between 4 hours and 2 days. No studies report whether the training was free or if the care home paid for it.

##### 3.5.3.1 Compatibility

In three studies of cognitive stimulation, it was apparent that the intervention fit into an existing scheme of group activity sessions led by care home staff, including memory training, physical exercise, cooking, or occupational therapy groups, which were available for the non‐intervention group [19, 53, 55]. This compatibility was explicitly stated in the interviews with intervention deliverers in the study of sports‐based reminiscence [50]; “the respondents had found no real problems in implementing the work following the training.”

##### 3.5.3.2 Structural Characteristics—Work Infrastructure

A significant barrier across three studies was staff availability and time to deliver the intervention. One study of MAKS reported the need to retrain a staff member to deliver the intervention due to attrition [54]. Staff taking part in a study of reminiscence therapy reported that they had a lack of time to plan for and carry out reminiscence activities [48]. This was also the case for staff running sports‐based reminiscence sessions, where one staff member was coming back to the care home on their days off to run sessions [50]. However, interviews with staff highlighted that lack of time was always considered to be an issue in the care home, and this was not unique to delivering the intervention [50].

##### 3.5.3.3 Structural Characteristics—IT Infrastructure

The study of sports‐based reminiscence reported an online forum for staff across care homes in a network to share information. However, many staff were unable to access this from the care home IT systems [50].

##### 3.5.3.4 Culture—Recipient‐Centredness

Two studies referred to the culture within the care home setting and how this impacted intervention delivery. Staff delivering the sports‐based reminiscence reported that the intervention was compatible with and enabled a person‐centred approach to care [50]. However, in another study of reminiscence therapy, the authors reported a “lack of recognition of the importance of residents' social and emotional needs”, with staff focussing on “task‐oriented” work and physical wellbeing rather than holistic care and psychological wellbeing [48]. This highlights how psychosocial interventions and a person‐centred approach to care may or may not be priorities (CFIR construct: Relative priority).

##### 3.5.3.5 Relational Connections

This construct relates to relationships and networks within the care home supporting implementation. Notably, only one paper referred to within‐care home peer support and transfer of knowledge [50], stating that staff had been able to pass on the training in sports‐based reminiscence to others in their home. This is in contrast with the seven papers reporting that there was support and knowledge exchange from external partners [19, 48, 50, 51, 52, 53, 54].

#### 3.5.4 Individuals

This domain relates to individuals' characteristics and roles in implementing the intervention. The intervention deliverers and recipients were the most prominent themes.

##### 3.5.4.1 Innovation Deliverers

Four studies referred to the skills and capability of the staff delivering the intervention [48, 49, 50, 51]. Staff reflected on their varying confidence levels based on previous experience and the associated need for training and support [50], and reported that taking part in the interventions had improved their interactions with residents with dementia [48, 51]. Two studies reported on the staff's motivation to take part and their perceived acceptability of the intervention [48, 50].

##### 3.5.4.2 Innovation Recipients

None of the studies gathered qualitative data from the people with dementia or their carers participating in the intervention. Staff members reported participants' levels of engagement in three studies [48, 51, 55], and attendance rates were used as a proxy for the acceptability of the intervention [19, 51].

##### 3.5.4.3 Implementation Facilitators

Five papers reported on the role of researchers in supporting the care homes by carrying out site visits, providing guidance and feedback, checking fidelity to the handbook and materials [19, 48, 52, 53, 54].

##### 3.5.4.4 Implementation Leads

Two studies reported the role of an on‐site lead person to act as a link between the research team and the care home [48, 54]. While there was no specific feedback indicating that this link person facilitated the implementation process, it is worth noting that a link person is used in only a small number of studies. This observation reinforces findings that suggest external partners primarily drive the implementation efforts.

##### 3.5.4.5 Other Implementation Support

Two studies of reminiscence therapy discussed the role of family members in the intervention [48, 50]. There were mixed experiences, with some carers being “overprotective” and others not engaging. The need for guidance to engage family members was highlighted [50].

##### 3.5.4.6 High‐Level Leaders

One study of reminiscence therapy reported insufficient support from management [48]. This was perceived to be a barrier to full implementation, with staff wanting more discussion of reminiscence activities at staff meetings, management involvement in training and more praise from managers for the staff involved in reminiscence.

#### 3.5.5 Implementation Process

This domain relates to activities and strategies used to implement the innovation.

##### 3.5.5.1 Reflecting and Evaluation—Innovation

Eight papers used quantitative outcome measures to evaluate the impact of the intervention [19, 48, 49, 51, 52, 53, 54, 55], either on staff members or people with dementia, even when the study was not designed to detect change. Only three papers used qualitative methods, and none spoke to people with dementia and carers [48, 50, 51].

##### 3.5.5.2 Reflecting and Evaluation—Implementation

Eight studies gathered data relating to the success of implementation [19, 48, 50, 51, 52, 53, 54, 55], which we have described according to Proctor et al.’s taxonomy [43]. See the ‘Implementation outcomes’ section and Table 5 for more information.

**TABLE 5:** Implementation outcomes reported across studies.

Study	Implementation outcomes							
	Acceptability	Adoption	Appropriateness	Costs	Feasibility	Fidelity	Penetration	Sustainability
Clark, 2017 [50]	Qualitative data from staff interviews: Enthusiasm, staff “intended to continue to use the work in their homes”.		Qualitative theme from staff interviews: “Readily implementable and integrated into the life of the care home”.		Qualitative data from staff interviews: Time is limited, the intervention can be delivered flexibly.			
Coen, 2011 [55]	Staff's quantitative ratings of participants' interest, enjoyment, communication, and mood (ranging from 3.67 to 4.5/5)							
Graessel, 2011 [19]	Attrition: 4/50 PwD refused participation, and 7/50 did not meet minimum attendance. Attendance: Participants who completed the intervention missed on average 3% of intervention days.			Therapy costs are < €10 per day and person, with two therapists for 10 participants		Compliance with handbook: 97.5%		
Gudex, 2010 [48]	Qualitative interviews: 90% of staff considered reminiscence a “good work tool, 85% “would recommend it to other nursing homes.” Staff assessment of participant engagement: “Mostly positive” Attrition: 32% dropout rate for residents, 38% for staff (rate similar for control and intervention).	Difficulty in recruiting nursing homes was reported.			Reasons for low implementation: Lack of staff time to plan, poor management support, lack of staff interest.			
Kratzer, 2022 [54]	Attrition: 28/60 participants dropped out of intervention group.				COVID‐19 impacted intervention delivery.			
Lök,2019 [49]								
Luttenberger, 2012 [53]	Attendance: 3 refused therapy					Compliance with handbook: 97.5%		
Streater, 2016 [52]	Attendance: 55/68 people with dementia received full intervention.	Seven homes (50%) delivered the programme, and four homes (29%) were unable to deliver the programme.				Research staff provided outreach support and gave constructive feedback on adherence to intervention principles.		
Yamagami, 2012 [51]	Staff observation: “Participants looked cheerful”. Average attendance: 95.5%. Attrition: 0%.							

##### 3.5.5.3 Assessing Needs—Innovation Recipients

Four studies highlighted the importance of assessing the needs of people with dementia [19, 48, 50, 51], for example, related to their cognitive ability, interest in discussion topics, or life histories. Studies of reminiscence therapy and BAR reflected on the need for awareness of the emotional impact of reminiscence on participants [48, 51].

##### 3.5.5.4 Doing

This construct relates to implementation in small steps to trial intervention delivery. The study of sports‐based reminiscence reported on a pilot study scaled up with a broader network of care homes, with learnings from the pilot study implemented and the addition of the learning set meetings and online forum [50]. This also highlights adaptability in implementation processes (CFIR construct: Adapting).

### 3.6 The External Validity of the Sample

An additional code that was not within the CFIR framework was the perceived external validity of the study, which has important implications for learning about generalisability and broader implementation. All studies used care home staff as therapists, which is valid for nursing homes. In addition, five studies reported further on the external validity of the sample of care homes or participants.

One study made pen portraits of the care homes using information from the UK care home regulator, the Care Quality Commission, or CQC [50]. They ranged in size and level of service provision. However, they were not deemed representative of the sector because they all had ‘good’ reports. One study of MAKS reflected that the care homes were not randomly selected, but there was diversity in terms of urban and rural, and sheltered and open homes [54]. Another chose inclusion and exclusion criteria to reflect the clinical reality of people within nursing homes, for example, by including patients with poor cognitive function and challenging behaviour [19]. A study of reminiscence therapy included all residents in the reminiscence activities (with and without dementia) to reflect the reality of mixed populations in care homes [48]. Finally, another study of reminiscence therapy reflected on their convenience sample in one care home only, which may not be representative or generalisable [49].

## 4 Implementation Outcomes

Table 5 summarises the spread of Proctor's implementation outcomes across the nine studies [43]. All but one study reported on intervention acceptability. In two studies, care home staff's perceptions of the intervention were collected via interviews [48, 50], which are largely positive, but reporting of the qualitative methods is poor, and these interviews could be biased. No studies gathered feedback from the intervention recipients, but three reported the level of participants' engagement, rated by staff members, which was positive overall [48, 51, 55]. Six studies reported varying levels of intervention attendance and attrition, which may also relate to the acceptability of the intervention [19, 48, 51, 52, 53, 54, 55]. Without in‐depth interviews with participants or staff who dropped out, the scope for exploring acceptability is limited.

Feasibility relates to how well staff were able to implement the intervention. This data was reported in three studies from interviews with staff, administrative data and researchers' reflections [48, 50, 54]. Interventions were not fully implemented in each site due to barriers, including the COVID‐19 pandemic, lack of time and support from management, and lack of staff interest.

Adoption, which relates to the intervention uptake at an organisational level, is addressed in two studies by reporting the recruitment or dropout rate of care homes [48, 52]. One study reported on the appropriateness and perceived fit of the intervention in existing systems, which was elicited through interviews with care home staff [50]. Only one study reported on the costs of the intervention, reported as cost per person per day [19].

Three studies reported on compliance with the manualised handbook or intervention principles, which was either self‐reported by intervention deliverers or reported through research team visits [19, 52, 53].

No studies carried out longer‐term follow‐ups with care homes; as such, none explored or reported on sustainability. One service evaluation did report that some care homes had followed up with a longer‐term programme of CST during the study period [51], and staff in the formative evaluation of sports‐based reminiscence reported that they hope to continue using the intervention in future [50], but it is unclear if either intervention extended beyond the study period.

## 5 Discussion

This review has highlighted many factors impacting implementation. A key facilitator was the design of an intervention, specifically a standardised manual or set of intervention resources. However, the importance of intervention adaptability to meet recipients' needs and fit the care home's workflows was highlighted. Studies demonstrated partnerships and connections with research teams, facilitating knowledge transfer through training and outreach support. However, short‐term research grants funded all but one study, emphasising a lack of long‐term funding, which is crucial for sustainable implementation.

A key barrier was the lack of staff time and availability, perhaps unsurprising given that care homes often operate with limited staffing capacity, against a backdrop of burnout, underfunding and staff attrition [80]. Studies reported a perceived lack of support from care home management, with successful delivery of a psychosocial intervention relying on individual staff members' commitment to a person‐centred approach, rather than this being led and supported by management. While cognitive stimulation and reminiscence therapy are widely supported in the NHS due to their inclusion in NICE guidelines and the Memory Services National Accreditation Programme (MSNAP) [81], there is limited external pressure and a lack of wider policy or practice guidelines to promote in the context of care homes.

Factors impacting implementation generally did not differ between cognitive stimulation and reminiscence approaches; however, identifying recipient needs was especially important for reminiscence therapy, more so than for cognitive stimulation, where studies report the need to gather a life history with the support of family members.

### 5.1 Comparison to Literature

Similar to previous reviews of intervention implementation in care homes using the CFIR, the outer setting is the least considered domain, and the inner setting is the most commonly considered [28, 82]. However, in this review, we found that knowledge exchange tended to run from the research team to the care homes rather than within or across care homes, with only one study reporting on this through action‐set meetings, online forums, and informal knowledge sharing [50]. This may not be sustainable and raises questions about what happens when the research funding period ends.

Our findings align with previous reviews of intervention implementation in care homes, which suggests that many factors are setting‐specific [11, 28]. Common facilitators to implementation include staff training and education, collaboration with family members, and improved perceptions and professional approaches to people with dementia. Challenges include reallocation of staff time, a conflict between the need to focus on the physical safety of people with dementia and their psychological wellbeing and a lack of organisational or managerial support [11, 28].

Two studies of reminiscence therapy did not state a precise intervention frequency [48, 50]. Additional information in the studies indicates that the therapies occurred more naturally and spontaneously in various settings and one‐to‐one sessions rather than solely in structured group settings. A previous review of the implementation of psychosocial interventions in care homes highlights the importance of a flexible approach [11], especially when staff may be short of time or resources. A programme of 24‐h cognitive stimulation developed for routine care and everyday interactions has recently been developed, which may enable a more flexible and adaptable approach [83].

### 5.2 Intervention Deliverer

We only included studies in which the intervention was delivered by care home staff, as we believe this to be a key component of successful, long‐term implementation. However, more than twice as many full‐text studies use the research staff to deliver the intervention compared to care home staff. This raises questions about the ecological validity of the studies and the missed opportunities to collect data on care home staff's perceptions of the intervention. Furthermore, this may be feeding into care home staff being excluded from dementia training [26].

CST was initially developed to be delivered by a research psychologist with the support of care home staff [15]. However, in many trials, the intervention was conducted solely by psychologists or researchers [84]. It is important to note that non‐specialists can also deliver CST, which is a crucial factor for its sustainable implementation [85]. MAKS was developed from the outset to be delivered by care home staff, and all three studies of MAKS in this review have examined the efficacy of programmes delivered by care home staff members and supported by the research team [19, 53, 54].

#### 5.2.1 Implementation Versus. Efficacy

Some of the studies in this review evaluate interventions already proven effective. However, the studies are designed as underpowered RCTs, focussing on collecting quantitative outcome data, perhaps at the expense of richer qualitative data about attitudes towards the intervention or the success of implementation. Only one study prioritised qualitative methods, but the quality of reporting and analysis was low [50]. Notably, no studies gathered qualitative feedback from people with dementia or their carers participating in the intervention. One study evaluated specific implementation strategies of training and outreach support, and we cannot assess the efficacy of these strategies because they were not tested against a control group [52].

### 5.3 Qualitative Data From People With Dementia

This review emphasises the need for more qualitative data from individuals with dementia and their families regarding their views, preferences, and experiences related to participating in psychosocial interventions in care homes. Post‐intervention interviews can be challenging for some people with dementia, who may have difficulty recalling their experiences, but it is important to include and maximise the potential of people with dementia in qualitative research [86]. For instance, a focussed ethnographic approach could involve observing engagement during activities and asking real‐time questions about participants' views and experiences [87]. This method goes beyond quantitative ratings from group leaders on participants' interest, enjoyment of communication, and mood during sessions, as employed in a study of CST in this review [55]. Additionally, various forms of communication, such as verbal, nonverbal, drawing, and writing, could be employed to help people with dementia express themselves and share their thoughts in ways that are tailored to suit their abilities [1, 87].

### 5.4 Strengths and Limitations

A key strength of this review is using a commonly used deductive framework to explore implementation and synthesis results. This framework facilitates a clear plan for synthesising complex data that can be compared across other studies and reviews [41]. However, nearly 40% of CFIR constructs had no coded data (18/48). This does not necessarily mean that the CFIR is irrelevant but reflects that many papers do not report implementation aspects. When they do, it is not done systematically [44].

No studies explored the effectiveness of implementation strategies, so the barriers and facilitators reported are perceived rather than proven. This is a common problem across studies exploring implementation [1, 82]. Future studies could consider hybrid designs or empirical testing of specific strategies.

We did not include ‘implementation’ in our search terms since terminology is variable, and many studies report on factors related to implementation without labelling the study as such [44]. This resulted in a heterogeneous set of study designs. However, to reduce heterogeneity, we limited them by the intervention deliverer, setting, population, and intervention type.

We only included group interventions in our screening, as this is where the most substantial evidence for cognitive stimulation lies [14]. Including studies employing a wider range of techniques, including individualised cognitive stimulation, may have allowed a broader exploration of delivery methods in care homes.

We categorised CST and MAKS as cognitive stimulation interventions, per the recent Cochrane review [14]. We also included the study of BAR, a multi‐modal intervention combining reality orientation, reminiscence therapy and daily activities under the umbrella of cognitive stimulation [51]. Whilst this was not considered within the Cochrane review, we felt that the study met the criteria for a cognitive stimulation intervention for this review due to its similarities to other cognitive stimulation interventions.

We did not exclude studies based on their quality rating. Overall, the quality of the included studies was low. Most information about implementation experiences will be found in qualitative data; the qualitative elements from the included studies were of low quality.

## 6 Conclusion

This is the first review to synthesise evidence on implementing cognitive stimulation and reminiscence therapy in care homes. The review highlights key barriers and facilitators to implementation that align with those previously identified. The review highlights the field's reliance on research staff to deliver interventions rather than training and involving care home staff in evaluating interventions. There is a pressing need for high‐quality implementation studies involving collaboration, consultation and co‐design with those who will deliver the intervention routinely and the people with dementia who will receive it.

## Conflicts of Interest

The authors declare no conflicts of interest.

## Supporting information

Supporting Information S1

## Data Availability

The data that support the findings of this study are available from the corresponding author upon reasonable request.
